# Spatial-Temporal Distribution, Morphological Transformation, and Potential Risk of Dissolved Inorganic Nitrogen in the Contaminated Unconfined Aquifer from a Retired Nitrogenous Fertilizer Plant

**DOI:** 10.3390/ijerph19138022

**Published:** 2022-06-30

**Authors:** Kunhua Yang, Dengdeng Jiang, Yun Chen, Jing Wei, Feiyang Xia, Wenyi Xie, Yan Zhou, Xuwei Li, Shaopo Deng

**Affiliations:** 1 Nanjing Institute of Environmental Science, Ministry of Ecology and Environment, Nanjing 210042, China; yangkunhua@nies.org (K.Y.); jiangdengdeng@nies.org (D.J.); chenyun@nies.org (Y.C.); weijing@nies.org (J.W.); feiyangxia28@163.com (F.X.); wenyixie@foxmail.com (W.X.); zhouyan@nies.org (Y.Z.); lixuwei@nies.org (X.L.); 2 Key Laboratory of Soil Environmental Management and Pollution Control, Ministry of Ecology and Environment, Nanjing 210042, China

**Keywords:** inorganic nitrogen, environmental behavior, transformation and transport, water quality, groundwater

## Abstract

The accumulation of nitrogen in groundwater in the industrial plots, especially the high ammonium, can result in a serious threat to the groundwater system in the urban area. This study monitored the dissolved inorganic nitrogen (DIN) of the polluted groundwater four times in one year in a retired nitrogenous fertilizer plant site with a production history of nearly 40 years, to analyze the spatial-temporal characteristics of DIN species (NH_4_^+^-N, NO_3_^−^-N, and NO_2_^−^-N) and the effects of groundwater environment on their transfer and transformation. The results showed that NH_4_^+^-N (<0.025 to 1310 mg/L) was the main DIN species (61.38–76.80%) with low mobility, whereas the concentration of NO_3_^−^-N and NO_2_^−^-N was 0.15–146 mg/L and <0.001–12.4 mg/L, accounting for 22.34–36.07% and 0.53–2.83% of total DIN, respectively. The concentration and proportion of NO_3_^−^-N and NO_2_^−^-N showed an upward trend with time, posing a threat to the safety of surrounding groundwater, and their high spatial-temporal variation was related to the morphological transformation and the transport. In the wet season, the pH and redox condition benefited the nitrification, and NO_3_^−^-N easily migrated from the deep soil solution to groundwater, hence the NO_3_^−^-N can be accumulated. Therefore, the analysis of species and behaviors of DIN in shallow groundwater is indispensable for environmental risk assessment.

## 1. Introduction

Nitrogen is an essential nutrient element in the biosphere, and nitrogen pollution in aquatic ecosystems is becoming a worldwide issue [1]. Notably, nitrogen pollution in soil and groundwater in the nonagricultural system can threaten groundwater safety in urban areas [2]. The contamination of nitrogen can cause environmental risks via the transport of nitrogen-polluted groundwater to surface water [3], and the transformation of NH_4_^+^-N into the cancer-causing NO_3_^−^-N and NO_2_^−^-N [4]. Another critical pathway is the upward transfer of greenhouse gases (such as NH_3_ and N_2_O) into the unsaturated zone and the atmosphere [5,6]. However, excessively high ammonia-nitrogen (NH_4_^+^-N) levels in the subsurface water have been less attracted attention, although its environmental risk in the retired nitrogen fertilizer plant site cannot be ignored. As reported by the previous study, in the retired nitrogenous fertilizer plant sites, the NH_4_^+^-N concentration in groundwater can be up to 3320 mg/L [7], which greatly exceeds the standard value (1.5 mg/L) of the worst quality according to the Chinese “Quality Standard for Ground Water” (GB/T14848-2017) [8]. On the other hand, the adsorption of NH_4_^+^-N into the soil can be up to 15,000 mg/kg [7], hence soil can be a polluted source that slowly releases NH_4_^+^-N to groundwater in the long term. Therefore, NH_4_^+^-N polluted groundwater in the retired nitrogenous fertilizer plant land is becoming a significant environmental issue, of which the monitoring and effective management should be raised greater concerns.

The NH_4_^+^-N is one of the major pollution components of some contaminated groundwaters, such as the landfill-leachate-contaminated groundwater [9], but its transport processes and transformation mechanism through aquifers are complex [10]. In contaminated groundwaters, movement of NH_4_^+^-N is retardative and may be approximately 0.25 times the average groundwater velocity [11], thus NH_4_^+^-N polluted groundwater can be retained underground and cannot be easily removed. Although denitrification is a dominated N removal mechanism in the phreatic water-saturated zone, the activities of denitrification are limited by a lack of organic-C or inorganic electron supplies [12]. It is widely recognized that the dissimilatory nitrate reduction to ammonium (DNRA) is an important process involving dissimilatory transformation of nitrate to ammonium [13]. For decades, it is considered that anaerobic ammonium oxidation (anammox) has a great influence on the nitrogen cycle and is an important process resulting in ammonium attenuation in the subsurface biosphere [13,14]. In general, the morphological transformation of inorganic nitrogen occurs throughout the microbial nitrogen cycle, and nitrate attenuation dominated by denitrification is the main pathway of N loss in the phreatic water-saturated zone [15].

On the other hand, the environmental behaviors of nitrogen can be affected by the groundwater environment. For instance, nitrogen is particularly sensitive to redox conditions because of its multiple valence electrons and species [16]. Under the condition of −381 mV < Eh < 193 mV and 6.84 < pH < 7.32, two types of nitrogen reduction can occur, i.e., DNRA and denitrification, whereas the nitrification as an oxidation pathway can be inhibited [17]. Moreover, the temporal variations of dissolved nitrogen concentration in shallow groundwater can be affected by seasonal rainfalls [18]. The nitrate-nitrogen (NO_3_^−^-N) in the soil solute of the vadose zone can be leached and migrate into shallow groundwater in the rainfall recharging process during the wet season. Furthermore, electrical conductivity (EC) and dissolved oxygen (DO) can reflect or control the total N and NO_3_^−^-N concentrations in unconfined groundwater [19]. Understanding the spatial and temporal variation of nitrogen pollution in groundwater and its controlling factors is imperative to explore the pollution levels and present a scientific strategy for risk management and control and pollution remediation.

Although many studies have explored the nitrogen levels and behaviors in the shallow groundwater, it still lacks the related investigation carried out in the retired nitrogenous fertilizer plant sites, which cannot be ignored for the sustainable development of cities. The main objective of this study was to show the spatial-temporal variation of different inorganic nitrogen in shallow groundwater and how it is controlled by the environmental factors in a contaminated retired nitrogenous fertilizer plant site. The aims of this study were to (1) obtain the spatial and temporal characteristics of DIN species and environment indicators and assess the groundwater quality based on the government standard; (2) analyze the impacts of the critical environment indicators (such as pH, ORP, available C, and rainfall) on the transformation among different DIN species. The findings in this study ought to provide an important observation of the environmental behaviors of nitrogen in the nonagricultural system and the indication of the potential for effective and sustainable management of contaminated groundwater in the unconfined aquifer.

## 2. Materials and Methods

### 2.1. Overview of the Study Area

**Figure 1:**
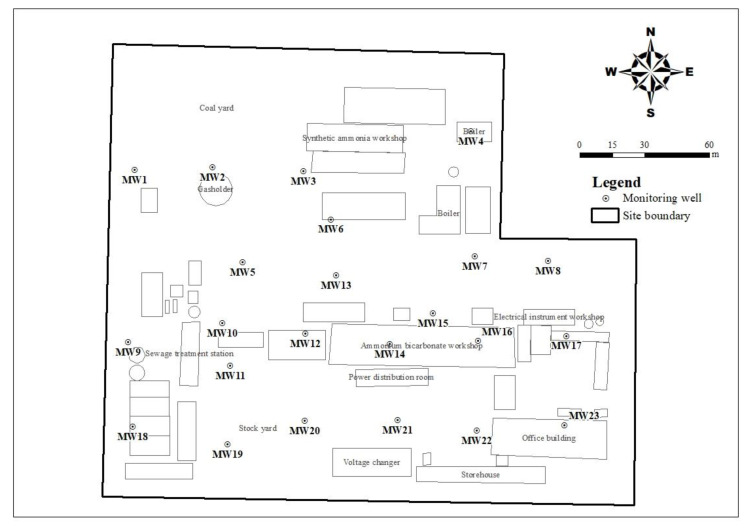
Layout plan of the nitrogenous fertilizer plant area and the sampling sites. “MW” indicates the monitoring well. The squares indicate the plant buildings. The circles indicate the other’s structures, such as the gas tank and factory chimneys.

The retired nitrogenous fertilizer plant site (Figure 1) selected as the study area has a production history of nearly 40 years. Its main products are ammonia, ammonium bicarbonate, and methanol, and its by-products are sulfur. The whole area of this plant site is about 73,000 m^2^, and the structures mainly included an ammonium bicarbonate workshop, synthetic ammonia workshop, methanol workshop, sewage treatment station, boiler room, coal yard, and storage yard. According to previous studies on retired nitrogenous fertilizer plant sites [7,20,21], the core production area, sewage treatment area, and storage yard may be the main areas that were contaminated with ammonia-nitrogen in groundwater.

The study area locates in northwest Jiangsu Province, China, belonging to a warm temperate semi-humid monsoon climate, with high temperatures and rainy in summer and frequent cold waves in winter. The annual average precipitation is about 900 mm, of which 70–83% of precipitation occurs from June to October, and the annual average temperature is about 15 °C. The landform of the study area belongs to the transition zone between the low mountains and hills in the southern margin of Shandong Province and the Huanghuai alluvial plain in the North China Plain and is about 300 m away from the Beijing-Hangzhou Grand Canal. The bedrock type is dolomite rock, and the shallow groundwater is stored in overlying Quaternary sediments. The terrain in the plot is relatively flat, roughly higher in the east and west area, but lower in the middle area. The historical stable water level is −1.85 to −3.69 m, with an average of −3.2 m and an annual variation of −1 to −2 m. The Quaternary pore phreatic water in the site is mainly distributed from −2 to −9 m.

### 2.2. Sampling and Analysis

In this study, 23 monitoring wells were set up to cover the potential pollution area of ammonia-nitrogen. According to the stratigraphic profile of the study area, the investigation depth of groundwater was about 13 m, to reach the roof of the first aquiclude, and the screening position of the sieve tube is set at −1.5 to −10 m. Groundwater samples were collected from 23 monitoring wells in the study area during the dry season (December 2020, January 2021) and the wet season (June and September 2021), using a Low Flow Pneumatic Groundwater Sampling Pump (Sample Pro, QED Environmental Systems Ltd. Dexter, MI, USA). The samples were collected and then transported to the laboratory and stored at approximately 4 °C. The groundwater quality parameters were measured on-site, including water temperature (T), pH, DO, oxidation-reduction potential (ORP), EC, and total dissolved solids (TDS). In the laboratory, inorganic nitrogen (NH_4_^+^-N, NO_3_^−^-N, and NO_2_^−^-N) and sulfate were determined by Spectrophotometry. Chloride was titrated by silver nitrate in a neutral condition. Heavy metals (Cu, Cr, Ni, Zn, and As) were analyzed by inductively coupled plasma mass spectrometry (ICP-MS). The determination of total organic carbon (TOC) in groundwater was using combustion oxidation nondispersive infrared absorption method in September 2021.

In this study, Excel 2013 software (Microsoft Corporation, Redmond, WA, USA). is used for statistical calculation of groundwater detection data, IBM SPSS statistics 20 software (IBM Corporation, Armonk, NY, USA) is used for correlation analysis, Origin 2018 software (OriginLab Corporation, Northampton, NC, USA) is used to draw relevant data maps, and ArcGIS 10.5 software (Environmental Systems Research Institute, Inc., Redlands, CA, USA) is used to draw spatial interpolation.

## 3. Results and Discussion

### 3.1. Concentration and Distribution of Dissolved Inorganic Nitrogen (DIN)

#### 3.1.1. Concentration of DIN Species and Groundwater Quality Assessment

**Figure 2:**
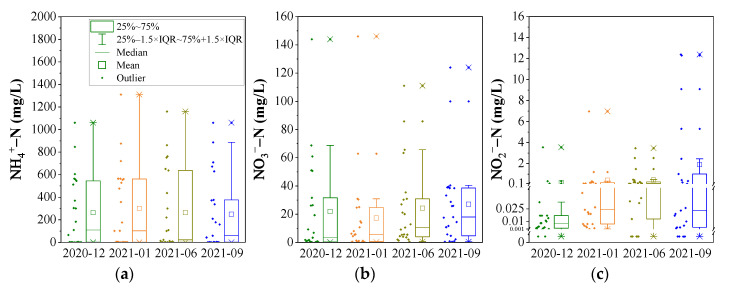
The box diagram of ammonia-nitrogen (**a**), nitrate-nitrogen (**b**), and nitrite-nitrogen (**c**) in groundwater.

As shown in Figure 2a, the concentration of NH_4_^+^-N was from <0.025 mg/L to 1310 mg/L. The average of NH_4_^+^-N in a sampling period was 262.47 mg/L, 300.58 mg/L, 263.17 mg/L, and 247.93 mg/L, in December 2020, January, June, and September 2021, respectively, with a median value of 110.0 mg/L, 102.0 mg/L, 22.0 mg/L, and 58.4 mg/L. The average value of NH_4_^+^-N concentration was much higher than that of the median value in each group of data, which indicates that several data points of average NH_4_^+^-N are abnormally higher. Meanwhile, it can be found that the temporal distribution of average NH_4_^+^-N concentration is related to that of the maximum value. Considering the data of NH_4_^+^-N concentration does not conform to the normal distribution, the median value is used to compare the temporal trend of NH_4_^+^-N concentration. Obviously, NH_4_^+^-N concentration shows a higher value in the dry season, but a lower value in the wet season.

The concentration of NO_3_^−^-N and NO_2_^−^-N was 0.15–146 mg/L and <0.001–12.4 mg/L (Figure 2b,c), which was lower than that of NH_4_^+^-N by about one and two orders of magnitude, respectively. Similar to NH_4_^+^-N, the data of NO_3_^−^-N and NO_2_^−^-N show a non-normal distribution with a right deviation pattern. Comparing the temporal variation of median value, NO_3_^−^-N and NO_2_^−^-N concentration display an increasing trend with time, except for a declining variation of NO_2_^−^-N in September 2021. The median value of NO_3_^−^-N concentration was 3.4 mg/L, 5.7 mg/L, 10.5 mg/L, and 18.0 mg/L, and that of NO_2_^−^-N concentration was 0.008 mg/L, 0.024 mg/L, 0.085 mg/L, and 0.023 mg/L, in December 2020, January, June, and September 2021, respectively.

Compared with the standard value of categories V (the worst quality) according to the “Quality Standard for Ground Water” (GB/T14848-2017) [8], for four sampling periods, the contaminated proportion of NH_4_^+^-N was 65.2%, 73.9%, 69.6%, and 69.6%, that of NO_3_^−^-N was 26.1%, 17.4%, 30.4%, and 34.8%, and that of NO_2_^−^-N was 0%, 4.3%, 0%, and 17.4%, respectively. Compared with the standard value of categories III, the contaminated proportion of NH_4_^+^-N was 82.6%, 87.0%, 87.0%, and 82.6%, that of NO_3_^−^-N was 34.8%, 26.1%, 43.5%, and 47.8%, and that of NO_2_^−^-N was 4.3%, 8.7%, 13.0%, and 26.1%, respectively. In these terms of contaminated proportion, NH_4_^+^-N exceeds the standard most seriously and changed little, but NO_3_^−^-N and NO_2_^−^-N show the highest pollution level in September 2021.

To scientifically reflect the levels of potential pollutants (NH_4_^+^-N, NO_3_^−^-N, NO_2_^−^-N, Cl^−^, SO_4_^2−^, Cu, Cr, Ni, Zn, and As) and comprehensive pollution, two methods were used to assess groundwater quality, including the Grading method and the Nemerow index method, of which the detail calculated steps were described in the reference [22]. The groundwater quality for all samples was classified into I–V categories according to the “Quality Standard for Ground Water” (GB/T14848-2017) [8], and the I–V categories was assigned to the *F_i_* value as 0, 1, 3, 6, and 10, respectively. The *F_i_* value for ten pollutants was used to calculate the *FI* value, the comprehensive score for all samples, and the *FI_region_* value was calculated using the *FI* value to obtain a comprehensive score for all potential pollutants in the study area [22]. According to the *FI* value (0–10), the Grading method rated the groundwater quality into five grades, which were “Excellent” (*FI <* 0.80), “Good” (0.80 ≤ *FI* ≤ 2.50), “Moderate” (2.50 ≤ *FI* ≤ 4.25), “Bad” (4.25 ≤ *FI* ≤ 7.20), and “Very bad” (*FI* > 7.20). The Nemerow index method was based on the single factor pollution index (*P_i_*), which value was the quotient of pollutant concentration divided by the limited standard value of categories III. Next, the *P_i_* value was used to calculate the *PI* and *PI_region_* value, and the formulations were as the description in the previous study [22].

**Figure 3:**
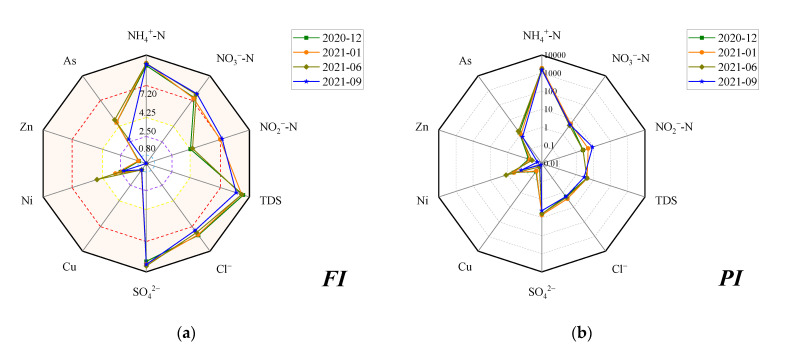
The comprehensive scores for potential pollutants in groundwater: *FI* values by the Grading method (**a**) and *PI* (**b**) values by the Nemerow index method.

The *FI* and *PI* values for ten pollutants are displayed in Figure 3. The order of the average of *FI* value followed as: SO_4_^2−^ (9.27) > NH_4_^+^-N (9.18) > TDS (9.16) > Cl^−^ (7.96) > NO_3_^−^-N (7.68) > NO_2_^−^-N (5.82) > As (4.26) > Ni (3.12) > Cu (0.74) > Zn (0.37). Expect for Cu and Zn always locating in the “Very Good” grade, Ni ranging from “Good” to “Bad”, and other pollutants indicate a groundwater quality rating “Bad” to “Very bad”. The order of the average of *PI* value followed as: NH_4_^+^-N (1666.70) > SO_4_^2−^ (5.84) > NO_3_^−^-N (4.71) > NO_2_^−^-N (4.70) > TDS (3.91) > Cl^−^ (2.09) > As (1.13) > Ni (0.55) > Zn (0.04) > Cu (0.02). Similar to the *FI* value, the *PI* value reflects the enrichment of concerned pollutants, excluding Cu, Zn, and Ni. For four sampling periods, the *FI_region_* value was 7.75, 7.81, 7.86, and 7.68, and the *PI_region_* value was 1097.61, 1350.93, 1195.56, and 1094.20, respectively. Interestingly, the comprehensive assessment of groundwater quality reflects the variation of groundwater quality that is not getting worse, although the pollution level of NO_3_^−^-N and NO_2_^−^-N seems to increase with time.

#### 3.1.2. The Spatial Distribution Characteristics of DIN

**Figure 4:**
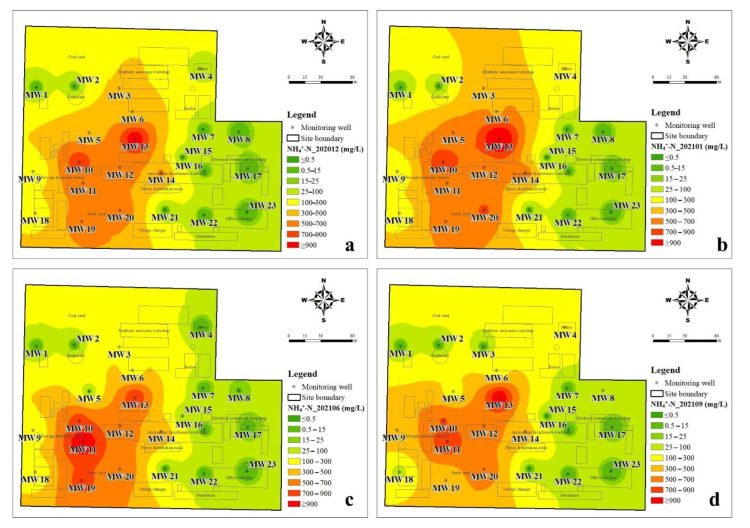
Interpolation diagram of ammonia-nitrogen in groundwater (**a**): in December 2020, (**b**): in January 2021, (**c**): in June 2021, (**d**): in September 2021.

**Figure 5:**
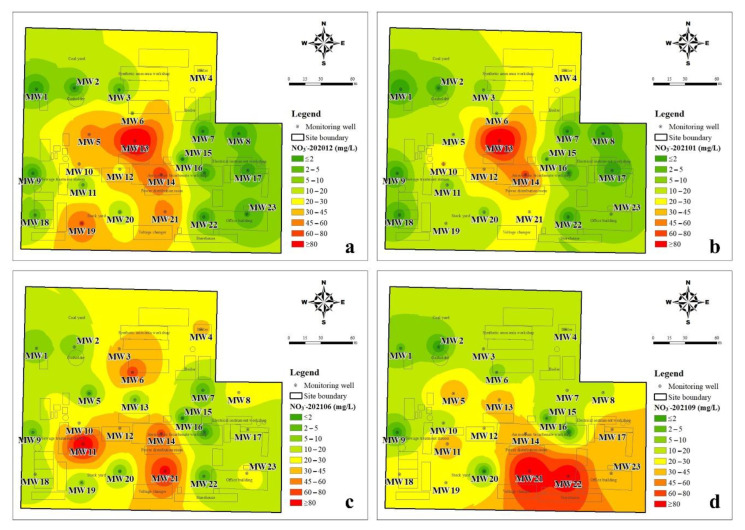
Interpolation diagram of nitrate-nitrogen in groundwater (**a**): in December 2020, (**b**): in January 2021, (**c**): in June 2021, (**d**): in September 2021.

**Figure 6:**
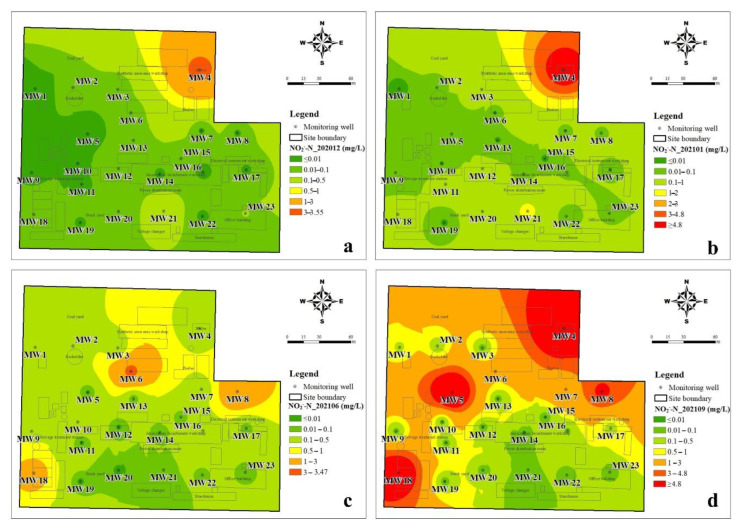
Interpolation diagram of nitrite-nitrogen in groundwater (**a**): in December 2020, (**b**): in January 2021, (**c**): in June 2021, (**d**): in September 2021.

The inverse distance weighting method was used to illustrate the spatial distribution of inorganic nitrogen in groundwater. It can be seen from Figure 4 that the spatial distribution of NH_4_^+^-N changes little during the four sampling periods and is closely related to the production function layout of the plant. The NH_4_^+^-N > 300 mg/L in groundwater was mainly located in the ammonium bicarbonate workshop, synthetic ammonia workshop, sewage treatment station, and storage yard. The groundwater with a high NH_4_^+^-N value (>700 mg/L) was mainly located in the ammonium bicarbonate workshop, sewage treatment station, and storage yard. As shown in Figure 5, the spatial distribution of NO_3_^−^-N in the dry season was similar to that of NH_4_^+^-N, but it changed significantly in the wet season. In the dry season, the groundwater level was low and groundwater flow was slow, thus the migration and transformation of DIN were lower. NO_2_^−^-N is an unstable component in groundwater and is the intermediate product of the nitrification or denitrification process. The temporal and spatial distribution of NO_2_^−^-N in the study area changed greatly, but the NO_2_^−^-N concentration was relatively high in the northeast of the study area during the all-sampling periods (Figure 6). The obvious accumulation of NO_2_^−^-N in the wet season may be related to a higher reduction rate of nitrate than that of nitrite [23].

### 3.2. Indication of pH and ORP Condition

**Figure 7:**
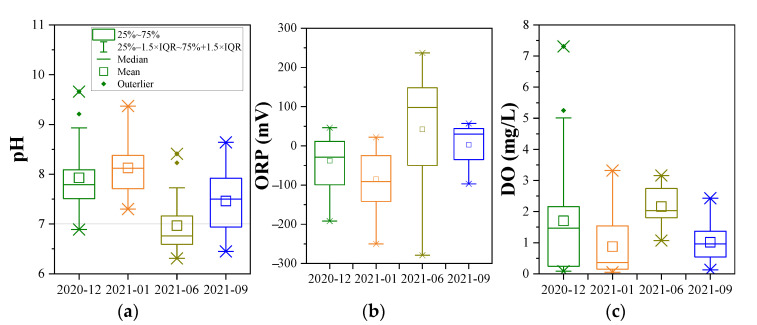
Variation of pH (**a**), ORP (**b**), and DO (**c**) of groundwater.

Nitrogen is essential for all living organisms, and ammonium and nitrate are two of the bioavailable forms for most organisms to grow [24]. As the consequence of and the constraint on oxidation-reduction reactions, the ORP reflects the habitats for microorganisms [25]. The combination of pH and ORP is helpful to understand the stable product or valence state of nitrogen in the groundwater system. According to the results of chemical thermodynamics obtained under standard conditions (25 °C and 1 atm) [17], when the ORP ranges from −500 mV to +300 mV at pH < 9.23, ammonium ion (NH_4_^+^) is the most abundant species of DIN in groundwater, whereas nitrate ion (NO_3_^−^-N) is dominated under oxidizing conditions (ORP > 400 mV). In this study, the groundwater temperature varied from 12.5–23.2 °C, of which the impact on the transformation of DIN is usually in reaction rates but not reaction direction, hence this ORP–pH relationship can be applied to understand the behaviors of DIN in groundwater. In the study area, pH was from 6.31 to 9.66, and ORP was from −279 mV to 237 mV (Figure 7). According to the range of pH and ORP, NH_4_^+^ is the dominant species in according to the stability field of groundwater for DIN [17].

On the other hand, the change in pH and ORP may account for a variety of concentrations of NH_4_^+^-N and NO_3_^−^-N. Among different species of inorganic nitrogen, NH_4_^+^-N is an electron donor in nitrification, and NO_3_^−^-N is an electron acceptor in denitrification. The preference for redox species in oxidation reactions is DO > nitrate > sulfate, which is referred to as the terminal electron-accepting processes [9]. Under the high DO condition, NH_4_^+^-N is easily oxidated to NO_2_^−^-N and NO_3_^−^-N through nitrification, and the nitrification can occur in a large range of DO, i.e., 0.3–4 mg/L [26]. On the other hand, lower ORP conditions are favorable for denitrification to occur, being DO < 2 mg/L and ORP < 100 mV reported in the previous study [27]. In this study, the range and distribution of pH, ORP, and DO were very different each month, which indicates the occurrence of DIN transformation in groundwater. Although the correlation between ORP and DO was weak, their temporal variation was very similar, that was lower in the dry season and higher in the wet season. In the dry season, ORP and DO for most groundwater samples were low (<0 mV and <2 mg/L, respectively), which indicates that denitrification may prevail instead of nitrification, and this condition is beneficial to the removal of NO_3_^−^-N from groundwater [27]. The higher ORP and DO condition in the wet season benefits the nitrification that NH_4_^+^-N easily transforms to NO_2_^−^-N and NO_3_^−^-N, hence the NO_3_^−^-N can be accumulated [28].

### 3.3. Impact Factors of the Transfer and Transformation of DIN

#### 3.3.1. The Transformation of DIN and Correlation Analysis

**Figure 8:**
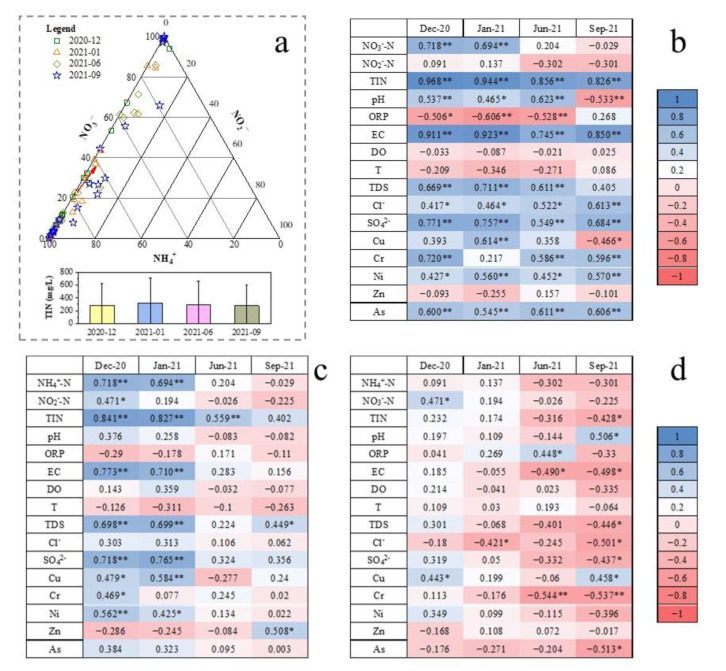
Temporal variation of DIN species and TIN concentration, and the Spearman correlation between inorganic nitrogen and environmental indicators (physicochemical parameters, major anion, and heavy metal). (**a**) The proportion of NH_4_^+^-N, NO_3_^−^-N, and NO_2_^−^-N, and the red arrow represents the direction of the change in the average proportion; the average TIN concentration, and the error bars represent the standard deviation. (**b**) the Spearman correlation between NH_4_^+^-N and environmental indicators. (**c**) the Spearman correlation between NO_3_^−^-N and environmental indicators. (**d**) the Spearman correlation between NO_2_^−^-N and environmental indicators. * *p* < 0.05, ** *p* < 0.01.

The concentration and proportion of different DIN species varied among four sampling periods (Figure 2 and Figure 8a). In general, NH_4_^+^-N was the major species of inorganic nitrogen in the groundwater of the study area, followed by NO_3_^−^-N, and then NO_2_^−^-N. In each sampling period, the average proportion of NH_4_^+^-N in inorganic nitrogen was 75.17%, 76.80%, 62.35%, and 61.38%, respectively, whereas that of NO_3_^−^-N was 24.30%, 22.34%, 36.07%, and 35.79%, and that of NO_2_^−^-N was 0.53%, 0.87%, 1.58%, and 2.83%. The proportion of NH_4_^+^-N makes decreasing in the wet season, whereas the proportion of NO_3_^−^-N shows an increase. The decreased NH_4_^+^-N in the wet season may be related to the recharging of rainwater that dilutes the NH_4_^+^-N concentration in groundwater, as well as the higher nitrification (see Section 3.2). Meanwhile, the total inorganic nitrogen concentration in groundwater (TIN) was different in each sampling period. On average, the TIN concentration was highest (318.17 ± 385.01 mg/L, average ± standard deviation) in January 2021, whereas that was lowest (276.84 ± 325.29 mg/L) in September 2021.

Since the change in groundwater environment can influence the transformation of DIN species, as well as the water-particle partition of DIN concentration, the Spearman correlation between DIN and several indicators of groundwater environment were analyzed. As shown in Figure 8b, NH_4_^+^-N was shown to have a significant positive relationship with TIN, EC, Cl^−^, SO_4_^2−^, Ni, and As in each period. Except September 2021, NH_4_^+^-N had a significant positive relationship with pH and TDS, but a negative relationship with ORP. The positive correlation between TIN and NH_4_^+^-N is because that NH_4_^+^-N was the major species of TIN; hence, the correlation between TIN and other parameters was not presented here. As the time increased, their correlation coefficients were decreasing, indicating that the impact of other DIN species on TIN concentration will be stronger. The significant positive correlation between NH_4_^+^-N and EC suggests that ionic ammonia (i.e., NH_4_^+^) is the main form of NH_4_^+^-N, instead of NH_3_·H_2_O. Furthermore, the positive correlation between NH_4_^+^-N and pH indicates that the proportion of NH_3_·H_2_O was rising with NH_4_^+^-N concentration. However, there was a negative correlation between NH_4_^+^-N and pH in September 2021, which may be caused by the heterogeneous mixing with rainwater. In this sampling period, a large amount of rainfall recharged the groundwater. Due to relatively low terrain in the middle part of the study area, in which NH_4_^+^-N concentration was higher, more rainwater recharging made pH lower. On the other hand, the significant positive correlation between NH_4_^+^-N and Cl^−^, as well as between NH_4_^+^-N and SO_4_^2−^, indicates that NH_4_Cl and (NH_4_)_2_SO_4_ are two components of pollution sources.

Furthermore, as shown in Figure 8c, the correlation coefficients indicate that the spatial distribution of NO_3_^−^-N was related to NH_4_^+^-N and NO_2_^−^-N in December 2020, whereas it appeared to become unaffected by them with time. The correlation in December 2020 may be related to the low flow of groundwater and the lacking exchange between surface water/rainwater and groundwater in the dry season. In the wet season, the recharge of rainwater disturbed groundwater, and then the NO_3_^−^-N can migrate far from NH_4_^+^-N due to higher mobility. Furthermore, the input from the vadose zone may be an important source of NO_3_^−^-N in the wet season (see Section 3.3.2), and the different NO_3_^−^-N inputs can increase the difference between the distribution of NH_4_^+^-N and NO_3_^−^-N. For NO_2_^−^-N, there was a significant difference between the dry season and the wet season (Figure 8d). No significant correlation between NO_2_^−^-N and most parameters was found in the dry season, except for a positive relationship with NO_3_^−^-N and Cu, and a negative relationship with Cl^−^. In the wet season, there was a significant negative correlation between NO_2_^−^-N and EC and Cr.

#### 3.3.2. Effect of Rainfall on the Transfer of Nitrate-Nitrogen

From the results in Figure 8, no significant correlation was found between NH_4_^+^-N and NO_3_^−^-N in the wet season. It is generally believed that NO_3_^−^-N in groundwater is unstable, whereas Cl^−^ is stable and relatively conservative. Based on the mass conservation, the proportion of rainfall recharging the groundwater can be calculated as [29]:

*f*_1_ = (*I*_w_ – *I*_2_)/(*I*_1_ – *I*_2_) (1)

*f*_1_ is the proportion of fresh rainwater, *I*_w_ is the Cl^−^ concentration of groundwater sample, *I*_1_ is the Cl^−^ concentration of rainwater endmember, and *I*_2_ is the Cl^−^ concentration of pollution source. Here, the Cl^−^ concentration of rainwater endmember used that of local rainfall in June 2020 which was 0.03 mg/L, and the Cl^−^ concentration of pollution source used that of MW11 monitoring well in June 2021 was 691 mg/L.

Δ*i* refers to the amount of variation for component *i*, due to the processes besides the physical mixing [30]. When Δ*i* > 0, the component *i* was accumulated, whereas Δ*i* < 0 represents that the component *i* was consumed.

Δ*i* = *i*_w_ − [*f*_1_ × *i*_1_ + (1 − *f*_1_) × *i*_2_] (2)

*f*_1_ is the proportion of fresh rainwater, *i*_1_ is the *i* concentration of rainwater endmember, and *i*_2_ is the *i* concentration of pollution source.

**Figure 9:**
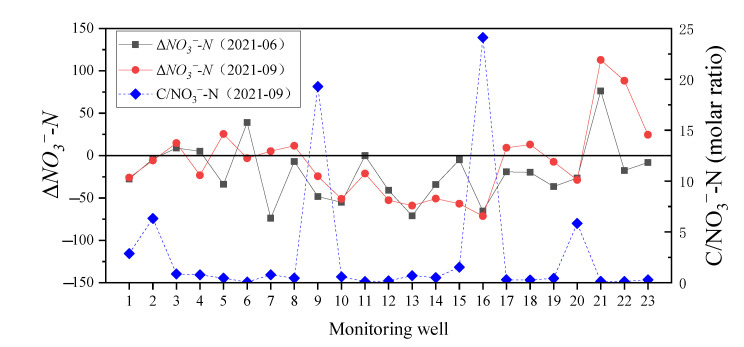
The Δ*NO_3_^−^-N* and C/N ratio of groundwater.

To calculate Δ*NO_3_^−^-N* in the wet season, the NO_3_^−^-N concentration of rainwater endmember used that of local rainfall in June 2020 which was 0.05 mg/L, and the Cl^−^ concentration of pollution source used that of MW11 monitoring well in June 2021 that was 111 mg/L. Meanwhile, the molar ratios of C/NO_3_^−^-N in September 2021 were calculated. The TOC in groundwater was used to calculate the C/NO_3_^−^-N ratio. The results of Δ*NO_3_^−^-N* and C/NO_3_^−^-N ratio were shown in Figure 9.

It can be seen that Δ*NO_3_^−^-N* for most groundwater samples in the wet season is lower than zero, especially in the middle part of study area, where the NH_4_^+^-N concentration is relatively higher. In other words, the accumulation of NO_3_^−^-N in groundwater is significant in the area that has low NH_4_^+^-N and relatively higher terrain. The contribution of atmospheric NO_3_^−^-N to groundwater in the study area was low and can be ignored because the NO_3_^−^-N concentration in rainfall was about 0.02–0.1 mg/L which is far less than that in groundwater. However, precipitation in the wet season had a great impact on the migration of NO_3_^−^-N. Previous studies have proved that NO_3_^−^-N can be accumulated in the deep soil solution of the vadose zone, which greatly affects the groundwater quality [31,32]. In this study area, the high concentration of nitrogen in groundwater most likely originated from the production activities of nitrogen fertilizer plants. When the nitrogen (in the form of NO_3_^−^-N, NH_4_^+^-N, and so on) transfers from the surface to the subsurface, NO_3_^−^-N can be accumulated in the deep soil solution of the vadose zone. Moreover, the groundwater depth can notably affect nitrogen concentrations in the shallow groundwater [18]. Therefore, NO_3_^−^-N can be leached from soil solute in the rainfall recharging process, as well as the rising groundwater level in the wet season, may lead to the migration of NO_3_^−^-N in the vadose zone to groundwater, thus increasing the concentration of NO_3_^−^-N in groundwater.

On the one hand, there may be microbial-mediated NO_3_^−^-N consumption in groundwater, including denitrification and dissimilatory reduction [33]. Denitrifying bacteria are essentially ubiquitous in the subsurface [34], and the occurrence of denitrification can be found in a large range of DO (0.2–4 mg/L, more favorable in DO < 2 mg/L) and pH (5.5–8) [35]. Additionally, the DNRA may be an important pathway for the removal of NO_3_^−^-N, which can account for 40–63% of the total biochemical consumption of NO_3_^−^-N [36], and C/NO_3_^−^-N ratio is an important index to estimate the occurrence of DNRA. The available carbon and the reducing conditions benefit the occurrence of DNRA, which is more favorable than denitrification when C/NO_3_^−^-N > 12 [37]. In this study, the C/NO_3_^−^-N ratios for most samples in September were lower than 12. In two monitoring wells (MW9 and MW16), C/NO_3_^−^-N > 12 in groundwater indicated that the DNRA may contribute to the consumption of NO_3_^−^-N. However, the DNRA is not conducive to the removal of N from groundwater due to the retaining of N as the form of NH_4_^+^-N. Therefore, denitrification is the potential important pathway for the removal of NO_3_^−^-N from groundwater in the study area.

### 3.4. The Temporal Tend of DIN in the Monitoring Wells

**Figure 10:**
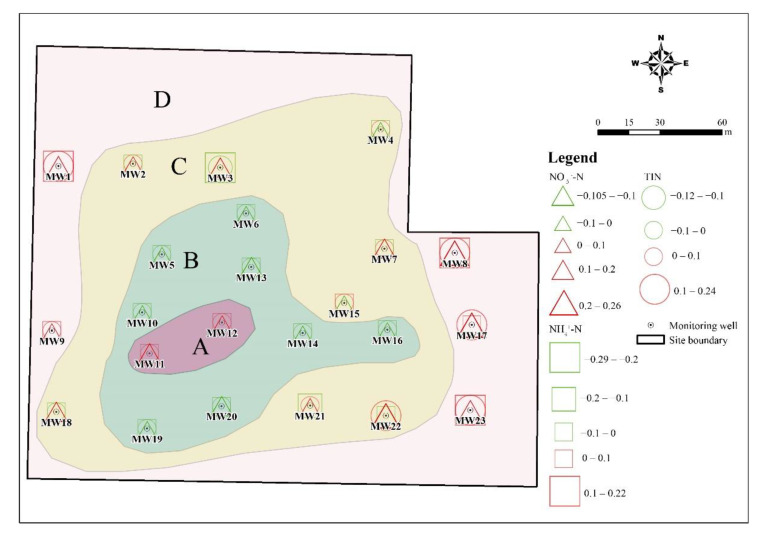
Map showing the temporal trends of DIN species in the monitoring wells. The triangle, square, and circle represent the estimated slopes of log_10_ NO_3_^−^-N, NH_4_^+^-N, and TIN, respectively. Green color indicates a negative slope, whereas red color indicates a positive slope.

The spatial distribution of DIN species showed very differently due to seasons. The linear regression has been developed to estimate trends in every single well within a year. The concentration of NO_3_^−^-N, NH_4_^+^-N, and TIN has been processed with a logarithm. The slope coefficients of these linear regressions were variable and reflect the degree and direction of the transformation of DIN species [38]. As shown in Figure 10, the slope coefficients varied spatially and can be divided into four-zone A–D. In zone A and zone D, the NO_3_^−^-N, NH_4_^+^-N, and TIN concentrations had been rising obviously. On the contrary, the DIN concentration declined in zone B. Zone C was a transitional zone and mainly characterized by the increased NO_3_^−^-N and the decreased NH_4_^+^-N. Both upward and downward trends are shown for different DIN species in each well, and this phenomenon is also shown in the previous study [39]. Although it is difficult to evaluate the general trend in the whole study area, the temporal tends of DIN in the monitoring wells display an obvious spatial distribution.

## 4. Conclusions

In this study, three species of dissolved inorganic nitrogen (NH_4_^+^-N, NO_3_^−^-N, and NO_2_^−^-N) were analyzed in groundwater that was collected from 23 monitoring wells in a retired nitrogenous fertilizer plant site. The sampling periods were four times in one year, and the spatial and temporal distribution of DIN species and their impact factors were discussed. The concentration of NH_4_^+^-N was from <0.025 mg/L to 1310 mg/L, and that of NO_3_^−^-N and NO_2_^−^-N was 0.15–146 mg/L and <0.001–12.4 mg/L, respectively. The spatial distribution of NH_4_^+^-N changes little, and the groundwater with a high NH_4_^+^-N value (>700 mg/L) was mainly located in the ammonium bicarbonate workshop, sewage treatment station, and storage yard. The spatial distribution of NO_3_^−^-N was similar to that of NH_4_^+^-N in the dry season but changed significantly in the wet season due to relatively higher groundwater level and flow rate. Although the distribution of NO_2_^−^-N changed significantly, the NO_2_^−^-N concentration was kept at a high value in the northeast of the study area. The results of the groundwater quality assessment suggested that NH_4_^+^-N, NO_3_^−^-N, NO_2_^−^-N, TDS, Cl^−^, SO_4_^2−^, and As were the main pollutants in the study, and the groundwater quality was not getting worse with time. NH_4_^+^-N is the major species of dissolved inorganic nitrogen and accounted for 61.38–76.80% of TIN on average, and NO_3_^−^-N accounted for 22.34–36.07% of TIN. The DIN species prevailed by NH_4_^+^-N were related to the ORP and pH of groundwater, which was from −279 to 237 mV for ORP and was 6.31–9.66 for pH. In the wet season, the higher ORP and DO benefit the nitrification, as well as the migration from the deep soil solution in the vadose zone to groundwater, hence the NO_3_^−^-N was accumulated. The groundwater environment (pH, ORP, and C/NO_3_^−^) indicated that denitrification was the potential important pathway for the removal of NO_3_^−^-N, instead of DNRA. The temporal trend of NO_3_^−^-N, NH_4_^+^-N, and TIN concentrations in each well can be divided into four-zone, indicating the spatial variation of DIN transformation.

The results in this study highlight the environmental risk of groundwater in retired nitrogenous fertilizer plant sites and demonstrated the morphological transformation of DIN that is affected by the groundwater environment. In a word, the spatial-temporal variation of DIN indicates that not only NH_4_^+^-N is the important pollutant in the groundwater in retired nitrogenous fertilizer plant sites, but also the NO_3_^−^-N and NO_2_^−^-N can be accumulated, transferred, and cause environmental risks. The groundwater environment (pH, ORP, and C/NO_3_^−^) is favorable for the removal of NO_3_^−^-N via the denitrification, indicating that the Monitored Natural Attenuation (MNA) may be practicable. However, the different patterns of DIN transformation, i.e., four-zone in Figure 10, suggest that more attention should be paid to the monitoring of DIN around the retired nitrogenous fertilizer plant sites, although it may not show the pollution at present. Further research is needed to quantify the transformation by combining the isotopes and microbiological methods.

## Data Availability

Not applicable.
